# Therapeutic Hypothermia and the Risk of Hemorrhage: A Systematic Review and Meta-Analysis of Randomized Controlled Trials

**DOI:** 10.1097/MD.0000000000002152

**Published:** 2015-10-30

**Authors:** Chih-Hung Wang, Nai-Chuan Chen, Min-Shan Tsai, Ping-Hsun Yu, An-Yi Wang, Wei-Tien Chang, Chien-Hua Huang, Wen-Jone Chen

**Affiliations:** From the Department of Emergency Medicine, National Taiwan University Hospital Yunlin Branch, Douliu City, Yunlin County (C-HW), Graduate Institute of Clinical Medicine, College of Medicine, National Taiwan University, Zhongzheng Dist., Taipei City (C-HW), Department of Emergency Medicine, Tao Yuan General Hospital, Ministry of Health and Welfare, Taoyuan Dist, Taoyuan City (N-CC), Department of Emergency Medicine, National Taiwan University Hospital and National Taiwan University College of Medicine, Zhongzheng Dist., Taipei City (M-ST, A-YW, W-TC, C-HH, W-JC), Department of Emergency Medicine, Taipei Hospital, Ministry of Health and Welfare, Xinzhuang Dist., New Taipei City (P-HY); and Department of Emergency Medicine, Lotung Poh-Ai Hospital, Luodong Township, Yilan County, Taiwan (R.O.C.) (W-JC).

## Abstract

Current guidelines recommend a period of moderate therapeutic hypothermia (TH) for comatose patients after cardiac arrest to improve clinical outcomes. However, in-vitro studies have reported platelet dysfunction, thrombocytopenia, and coagulopathy, results that might discourage clinicians from applying TH in clinical practice. We aimed to quantify the risks of hemorrhage observed in clinical studies.

Medline and Embase were searched from inception to October 2015.

Randomized controlled trials (RCTs) comparing patients undergoing TH with controls were selected, irrespective of the indications for TH. There were no restrictions for language, population, or publication year.

Data on study characteristics, which included patients, details of intervention, and outcome measures, were extracted.

Forty-three trials that included 7528 patients were identified from 2692 potentially relevant references. Any hemorrhage was designated as the primary outcome and was reported in 28 studies. The pooled results showed no significant increase in hemorrhage risk associated with TH (risk difference [RD] 0.005; 95% confidence interval [CI] −0.001–0.011; I^2^, 0%). Among secondary outcomes, patients undergoing TH were found to have increased risk of thrombocytopenia (RD 0.109; 95% CI 0.038–0.179; I^2^ 57.3%) and transfusion requirements (RD 0.021; 95% CI 0.003–0.040; I^2^ 0%). The meta-regression analysis indicated that prolonged duration of cooling may be associated with increased risk of hemorrhage.

TH was not associated with increased risk of hemorrhage despite the increased risk of thrombocytopenia and transfusion requirements. Clinicians should cautiously assess each patient's risk-benefit profile before applying TH.

## Abstract 2

Supplemental Digital Content is available in the text

## INTRODUCTION

Cardiac arrest is a common and lethal emergent condition.^1^ The significant mortality and neurologic disability following resuscitation may largely be attributed to the multisystem dysfunction caused by prolonged whole-body ischemia.^2^ Current guidelines^3^ recommend a 12- to 24-hours period of moderate therapeutic hypothermia (TH) (32–34 °C) for comatose adult patients with spontaneous circulation after cardiac arrest to improve survival and neurologic outcome.

The mechanisms of action of hypothermia are complex and not yet fully understood. The protective effect of TH may be explained by several mechanisms, including diminished cerebral metabolism,^4^ reduced production of free radicals,^5,6^ decreased apoptosis and mitochondrial dysfunction,^7,8^ and attenuated immune and inflammatory responses.^9^

Because of its diverse protective functions, TH has been studied widely in various patient populations in addition to patients following cardiac arrest,^10–12^ such as new-borns with hypoxic encephalopathy^13^ and patients sustaining traumatic brain injury,^14^ ischemic stroke,^15^ myocardial infarction,^16^ and bacterial meningitis.^17^ However, the efficacy of TH was not consistently confirmed across these studies.^10–17^ Additional investigations for identifying appropriate patient populations for TH may be needed.

The application of TH is not without risk.^18^ Various side effects have been observed that might discourage clinicians from applying this promising treatment. Hemorrhage is one of the complications that limits the widespread use of TH.^18^ Impaired platelet function, thrombocytopenia, and derangement of the coagulation cascade have been reported.^19–26^ Therefore, patients with bleeding diathesis have often been excluded from enrolment in TH clinical trials^11,12^ and from treatment with TH in real practice because of concern over the increased risk of hemorrhage.

Although the efficacy of TH for comatose patients with spontaneous circulation after cardiac arrest is currently under debate;^10–12^ clinicians might still consider applying this treatment, since there seems to be no other proven alternative that is more effective than TH for improving the dismal prognosis of these patients. A more complete understanding of the benefit-risk profile of TH may help clinicians make critical decisions regarding application of this treatment. However, previous meta-analyses^27,28^ that were restricted to a single indication for hypothermia enrolled only a few trials in analysis, which might have been too small to detect an association between TH and hemorrhage. Therefore, we conducted this systematic review and meta-analysis in an attempt to quantify the risks of hemorrhage in all patient populations receiving TH, irrespective of indication.

## METHODS

### Data Sources and Searches

We performed this meta-analysis in accordance with the Preferred Reporting Items for Systematic Reviews and Meta-Analyses guidelines.^29^ Two authors (CHW and NCC) independently searched Medline and Embase from their inception to October 2015 according to a prespecified protocol. Keywords of “hypothermia” and “cooling” were used for literature search, filtered with the term “randomized controlled trial” (RCT). The literature search was limited to human subjects; nonetheless, there were no restrictions on other aspects, including language, patient population, or year of publication. Abstracts from conferences, proceedings, or clinical trial registries were not included. Instead, we manually reviewed the bibliographies of relevant meta-analyses and reviews for references we may have missed during our primary search. This study was reviewed by the local Institutional Review Board and was exempt from requiring approval, since this research did not involve any human or animal subjects. The study protocol had not been registered in advance on accessible websites and was provided in the appendix.

### Study Selection

Two authors (CHW and NCC) independently scanned the titles and abstracts of all retrieved articles and selected those that were pertinent to this review. The following prespecified inclusion criteria were used: article reported an RCT, patients undergoing TH were treated by intentional reduction of core temperature below 36.0°C,^18^ control patients were treated using standard of care or controlled normothermia (ie, active temperature modulation with target temperature between 36.0 and 37.9°C),^18^ and the results included occurrence or absence of hemorrhage, coagulopathy, thrombocytopenia, or transfusion. Studies with hypothermia as part of a procedure, such as percutaneous coronary intervention for acute myocardial infarction, were included. Hemorrhage could be reported as “hemorrhage” in general or more specifically as intracerebral, pulmonary, or gastrointestinal hemorrhage. Abstracts were excluded.

After retrieval of articles reporting potentially relevant trials, 2 reviewers (CHW and NCC) independently assessed the eligibility of each study based on the inclusion criteria and resolved differing opinions by consensus or consultation with a third investigator (CHH).

### Data Extraction and Quality Assessment

Four authors (WTC, MST, PHY, and AYW) independently extracted data using a prespecified protocol. Each trial was reviewed independently by 2 of these 4 authors to ensure that the extracted data were correct. The extracted data included study design, study population characteristics, details of interventions, and types of outcomes measured.

The primary outcome of this meta-analysis was “any hemorrhage.” Other prespecified secondary outcomes included intracerebral/pulmonary/gastrointestinal hemorrhage, coagulopathy, thrombocytopenia, or any transfusion requirements (more specifically as transfusions for red blood cells, platelets, and plasma).

The Cochrane Collaboration tool for assessing risk of bias was adopted to evaluate the risk of bias for each RCT.^30^ The risk of bias for each trial was evaluated independently by 2 of the extraction authors (WTC, MST, PHY, and AYW). As our review was focused on the comparison of adverse effects between TH and control groups, specific questions were addressed when extraction authors assessed the risks of bias for blinding of outcome assessment and incomplete outcome data, including Are definitions of reported adverse effects given? Were the methods used for monitoring adverse effects reported? Discrepancies in assessment were resolved through discussions between the extraction authors or consultation with the supervising investigator (WJC).

### Statistical Analysis

Data synthesis and analysis were performed using the meta-package in the R 3.1.0 software (R Foundation for Statistical Computing, Vienna, Austria). A 2-sided *P* value ≤0.05 was considered statistically significant. Dichotomous outcomes from individual studies were collected to compute individual-study risk differences (RDs) with 95% confidence intervals (CIs). For studies with multiple intervention arms, all relevant treatment arms were grouped together by adding the sample sizes together and the event numbers together.^30^ Heterogeneity was evaluated using both the I^2^ statistic, with I^2^ ≥ 50% indicating a substantial level of heterogeneity, and the statistical test of heterogeneity, with a *P* value ≤0.05 indicating heterogeneity.^31,32^ As we pooled studies that differed in many clinical aspects, we chose to combine the effect estimates with a random effects model (DerSimonian-Laird method),^33^ irrespective of the value of the I^2^ statistic, or the significance of the heterogeneity test.

We performed subgroup analyses for primary outcome by prespecified covariates, including indication for TH, cooling method, cooling temperature, cooling duration, and temperature management of the control patients. The cooling temperature was divided into 3 categories based on the lowest target temperature of the TH patients as follows: mild (34.0–35.9 °C), moderate (32.0–33.9 °C), and deep hypothermia (<31.9 °C).^18^ The duration of cooling was divided into 3 categories, as follows: cooling duration <24 hours (including procedural hypothermia), 24 to 48 hours, and >48 hours. Temperature management of the control patients was divided into 3 categories as follows: standard of care, low-controlled normothermia (highest target temperature 36–37 °C), and high-controlled normothermia (highest target temperature between 37 and 38 °C).^34,35^ We tested these covariates for significance using univariate meta-regression analysis. We drew a funnel plot of the primary outcomes to evaluate publication bias.

## RESULTS

### Search Results and Description

**FIGURE 1:**
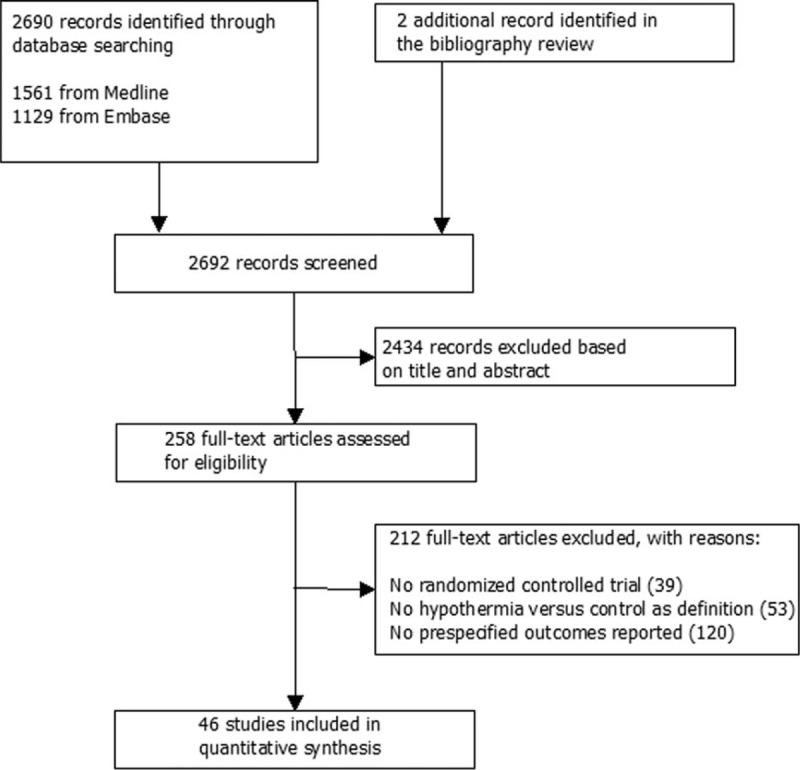
Flow diagram of the literature search.

The review process is shown in Figure 1. We identified 46 RCTs^10–17,36–73^ that included 7528 patients for the meta-analysis (Supplemental Table 1, http://links.lww.com/MD/A533). Indications for hypothermia varied greatly. Surface cooling was used in 27 studies, endovascular cooling in 15 studies, and 4 studies used both methods. Moderate hypothermia was applied in 35 studies, mild hypothermia in 6 studies, and deep hypothermia in 5 studies. Two studies^37,38^ used both 28 and 32 °C for the TH patients, which were classified as deep hypothermia. The duration of cooling ranged from several hours to 5 days. The methods of temperature management for control patients differed greatly; 9 studies did not clearly describe the methods used to control body temperature and were considered to be temperature management by standard of care.

**FIGURE 2:**
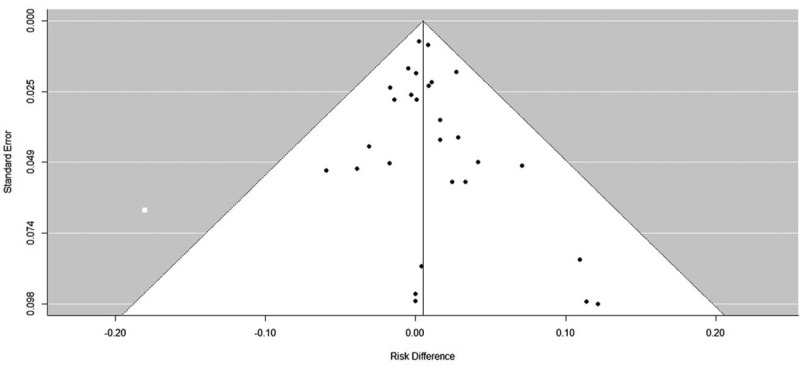
Funnel plot for the outcome of any hemorrhage.

As shown in Supplemental Table 2, http://links.lww.com/MD/A533, most studies could not blind healthcare personnel who assessed and reported outcomes. Furthermore, because definitions and follow-up periods of adverse outcomes were not uniformly and explicitly stated, there could be a high risk of bias. The funnel plot did not show obvious asymmetry, indicating that there might be no significant publication bias (Fig. 2).

### Synthesis of Quantitative Data

**FIGURE 3:**
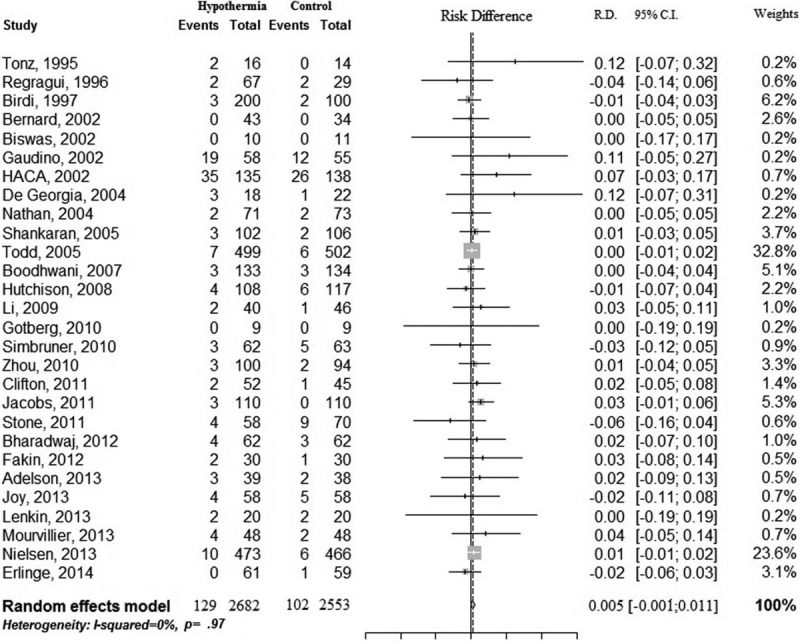
Forest plot of the summary effect estimates of any hemorrhage. CI = confidence interval, RD = risk difference.

The primary outcome of any hemorrhage was reported in 28 studies. Meta-analysis showed that TH did not significantly increase the risk of any hemorrhage (RD 0.005; 95% CI −0.001–0.011; I^2^ 0%) (Fig. 3) (Table 1). For secondary outcomes, TH also did not increase the risk of intracerebral, pulmonary, or gastrointestinal hemorrhage (Table 1). Although TH did not increase the risk of coagulopathy (RD 0.010; 95% CI −0.018–0.038; I^2^ 9.6%), TH seemed to increase the risk of thrombocytopenia (RD 0.109; 95% CI 0.038–0.179; I^2^ 57.3%). TH also increased the requirements for any transfusion (RD 0.021; 95% CI 0.003–0.040; I^2^ 0%), although for the transfusion of each type of blood component, there seemed to be no increased requirements (Table 1).

**TABLE 1:**
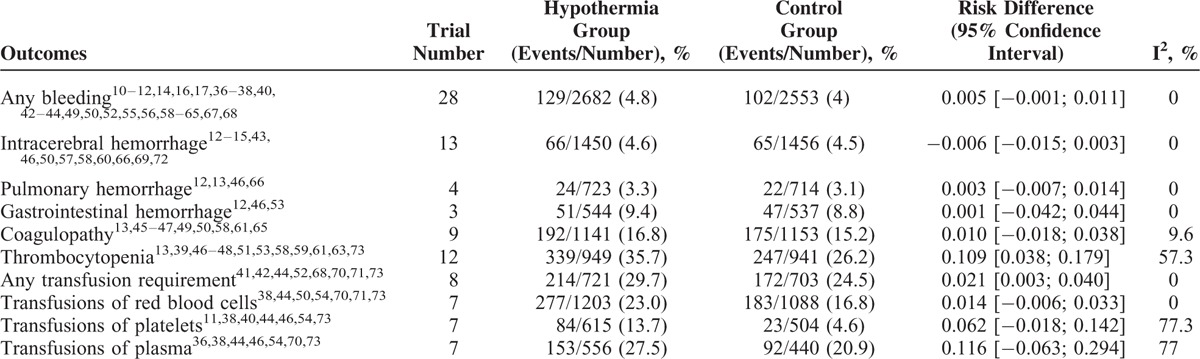
Summary of the Primary and Secondary Outcomes

Subgroup analysis showed that longer cooling duration might be associated with increased risk of any hemorrhage (*P* = 0.05) (Table 2).

**TABLE 2:**
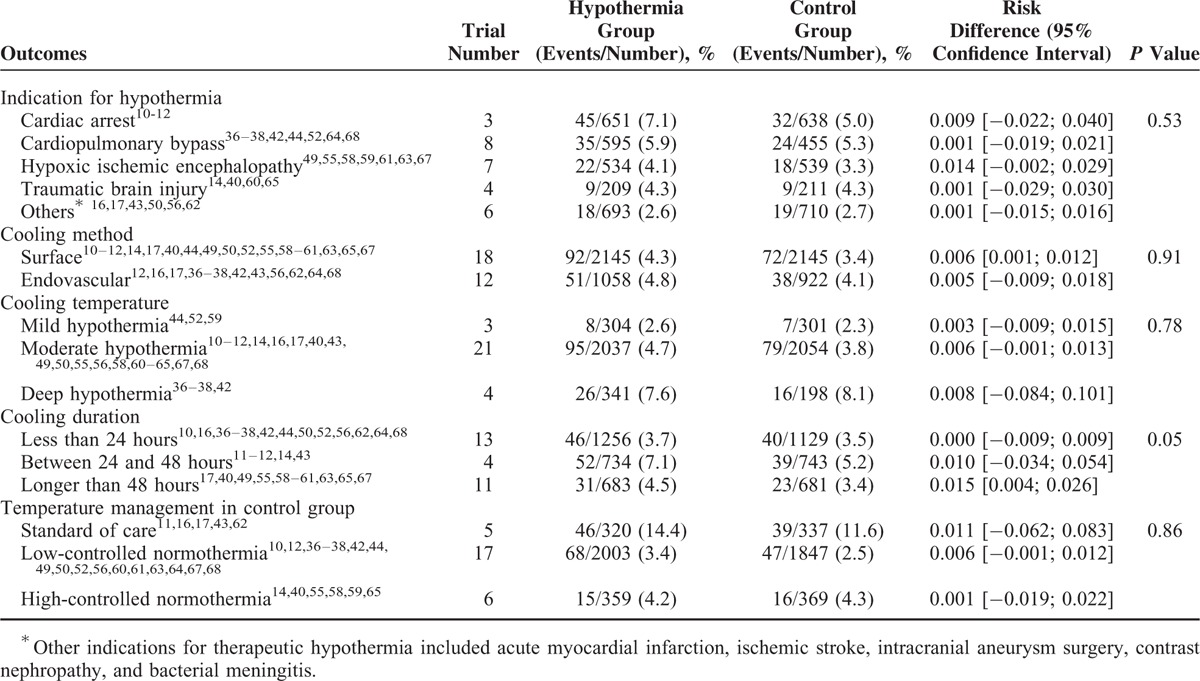
Summary of Subgroup Analysis

## DISCUSSION

This systematic review and meta-analysis did not find an increased risk of hemorrhage in patients treated with TH. Nonetheless, the risk of thrombocytopenia and transfusion requirements was increased for patients undergoing TH. Furthermore, a longer duration of cooling was also noted to be associated with increased risk of hemorrhage.

Two previous meta-analyses^27,28^ had reported that TH was not associated with increased risk of hemorrhage,^27,28^ transfusion requirements,^28^ and thrombocytopenia.^27^ However, these 2 meta-analyses^27,28^ mainly focused on patients sustaining cardiac arrest. The number of patients studied might be too small to detect the difference in adverse events, and the conclusions might be biased because observational studies were included.^27,28^ Therefore, we broadened the scope of indications for TH and thus included more patients and RCTs that studied TH, which allowed us to establish more robust associations between TH and adverse outcomes. Although there were no previous studies suggesting that the effect of TH would vary among different patient populations, and the subgroup analysis of our study also revealed that the risk of hemorrhage was not significantly different between patients with different indications for TH; the clinical and methodological heterogeneity were obvious. To be conservative, we chose a random-effects model to combine study results, even though statistical heterogeneity was found to be low.

There is no single definition or classification of TH. Various cut-off values have been proposed, with most studies referring to hypothermia as cooler than 35 or 36 °C. We used the definition proposed by Polderman and Herold,^18^ which referred to TH as the intentional reduction of a patient's core temperature to cooler than 36.0 °C. Based on this definition, many studies were excluded during the literature search and study selection, because they investigated unintentional hypothermia in operating rooms or accidental hypothermia in neonatal care. Studies comparing TH patients with control patients whose targeted temperature was between 35.0 and 36.0 °C were also excluded, because this temperature range would be considered hypothermic according to the definition of Polderman and Herold,^18^ instead of normothermic, which was assumed by the original studies.

The effects of hypothermia on coagulation have been studied mostly in vitro.^19–26^ Mild hypothermia (down to 35 °C) has no effect on platelet counts or function or the coagulation cascade.^19–26^ Temperatures cooler than 35 °C might have induced mild platelet dysfunction and sometimes a slight decrease in platelet count.^19–25^ When temperatures were cooler than 33 °C, other steps in the coagulation cascade, such as the synthesis and kinetics of clotting enzymes, appeared to be affected.^20–22,24^ Based on these in vitro observations, clinicians have tended to withhold TH for patients with a bleeding diathesis or withdraw TH for patients showing signs of hemorrhage. There have been few clinical studies focusing on the risk of hemorrhage in patients receiving TH. The perceived association of hypothermia with adverse clinical outcomes mostly originated from the lethal triad of hypothermia, acidosis, and coagulopathy, which is known to occur in trauma patients.^74,75^ However, it has also been reported that the stability of a clot formed before the initiation of TH is not affected by TH.^26^

Our meta-analysis found that TH was not associated with increased risk of any hemorrhage, including intracerebral/pulmonary/gastrointestinal hemorrhage. The risk of thrombocytopenia seemed to be increased by TH, while the risk of coagulopathy was not. Most of the RCTs in our meta-analysis used moderate hypothermia (32.0–33.9 °C). As reported by in-vitro studies, this temperature range might have had a greater effect on the platelet count or platelet function than on the coagulation cascade.^19–25^

Furthermore, although the risk of hemorrhage was similar between the TH and control patients, transfusion requirements were increased in TH patients. The increased transfusion requirements might be explained by the administration of prophylactic transfusions to TH patients by clinicians who noticed reductions in platelet counts. Nonetheless, the numbers of platelet transfusions administered to TH patients did not increase. The discrepancy in results between any transfusion requirements and platelet transfusions might be explained by the different RCTs that were used for each estimation of the effect of these 2 outcomes.

Subgroup analysis showed that the difference in risk of hemorrhage for surface and endovascular-cooling methods was not significant, although there have been concerns that initiation of hypothermia with the rapid infusion of crystalloids cooled to 4 °C might lead to acute hemodilution and subsequent coagulopathy.^76^ Subgroup analysis also revealed that the risk of hemorrhage might be associated with the duration of cooling instead of cooling temperature. In-vitro studies had shown that the extent of temperature decrease was associated with the extent of derangement in platelet function and coagulation cascade.^19–26^ Most studies (21/28, 75%) in our subgroup analysis used moderate hypothermia and fewer studies used mild or deep hypothermia, resulting in a subgroup statistical power that was insufficient for detecting differences in hemorrhage risks for the different categories of hypothermia.

A prolonged duration of cooling was found to be associated with increased risk of hemorrhage by subgroup analysis, which had not been previously reported. The optimal durations of cooling for TH applied to different indications are still being investigated. Longer duration of TH might lead to prolonged exposure to thrombocytopenic and greater chances of hemorrhage. However, cooling durations of longer than 48 hours mostly involved patients with traumatic brain injury or neonates with hypoxic ischemic encephalopathy, while cooling durations shorter than 12 hours mostly consisted of procedural cooling for patients suitable for elective operations. This difference in the composition of patient populations might be an important confounding factor that should be considered when interpreting our finding of increased risk of hemorrhage associated with prolonged cooling.

Increasing evidence from animal and human studies suggests that fever, irrespective of its cause, can directly and adversely affect the neurologic outcome of patients with various types of neurologic injury.^77^ The effect of controlled normothermia on risk of bleeding or coagulopathy has not yet been reported. Our subgroup analysis demonstrated that the risk estimates did not change significantly despite the great differences in temperature management of the control patients.

In summary, this systematic review and meta-analysis provided some supporting evidence that TH was not associated with increased risk of hemorrhage. However, TH was associated with increased risk of thrombocytopenia and transfusion requirements. In some studies,^78^ thrombocytopenia had been shown to be associated with increased cardiac mortality. Furthermore, it should also be emphasized that actively bleeding patients or patients with bleeding diathesis were excluded from most clinical trials studying TH, and generalization of our result should be cautiously weighed against the benefits of cooling. Nevertheless, the potential role of TH in the treatment of traumatic hemorrhagic shock is under investigation in clinical trials,^79^ which might shed light on this important issues more clearly.

## STUDY LIMITATIONS

First, there was tremendous clinical and methodological heterogeneity among the RCTs included in our study although the statistical heterogeneity was low. We chose a random effects model to combine the effect estimates, which might compensate for the presence of clinical and methodological heterogeneity to some extent. Second, follow-up periods of adverse outcomes were not explicitly reported across most included RCTs, which might cause attrition bias. Finally, the open assessment and lack of universal definitions for these adverse outcomes might cause detection and reporting bias.

## CONCLUSIONS

Although TH was associated with increased risk of thrombocytopenia and transfusion requirements, the risk of hemorrhage was not significantly associated with TH. Clinicians should cautiously assess the risk-benefit profile of each patient before applying TH.

## APPENDIX

Medline was searched according to the following strategy: (cooling) OR (hypothermia), Filters: Randomized Controlled Trial; Humans.

Medline was searched according to the following strategy: ((‘cooling’/exp or cooling and [embase]/lim) or (‘hypothermia’/exp or hypothermia and [embase]/lim)) and ‘randomized controlled trial’/de.
